# A Transcriptome‐Wide Association Study Identifies Candidate Susceptibility Loci and Genes for Lung Cancer Risk

**DOI:** 10.1002/cam4.71301

**Published:** 2025-10-16

**Authors:** Tianying Zhao, Jiajun Shi, Yaohua Yang, Dan Zhou, Jie Ping, Shuai Xu, Lili Xu, Jie Wu, Xiao‐Ou Shu, Ran Tao, Bingshan Li, Wei Zheng, Jirong Long, Qiuyin Cai

**Affiliations:** ^1^ Division of Epidemiology, Department of Medicine Vanderbilt Epidemiology Center and Vanderbilt University School of Medicine Nashville Tennessee USA; ^2^ School of Medicine University of Virginia Charlottesville Virginia USA; ^3^ Vanderbilt Genetics Institute Vanderbilt University Medical Center Nashville Tennessee USA; ^4^ School of Public Health and the Second Affiliated Hospital Zhejiang University School of Medicine Zhejiang Hangzhou People's Republic of China; ^5^ Department of Biostatistics Vanderbilt University Medical Center Nashville Tennessee USA; ^6^ Department of Molecular Physiology and Biophysics Vanderbilt University Medical Center Nashville Tennessee USA

**Keywords:** gene expression, lung cancer, susceptibility genes, transcriptome‐wide association study

## Abstract

**Background:**

Genome‐wide association studies (GWAS) have identified over 80 susceptibility loci for lung cancer risk. However, the genes underlying these associations remain largely unknown.

**Methods:**

We conducted a large transcriptome‐wide association study (TWAS) to identify lung cancer susceptibility genes. We leveraged gene expression data from lungs and 48 other tissue types and whole‐genome sequencing data from up to 706 samples of European ancestry in the GTEx (version 8) to build lung‐tissue and joint‐tissue gene expression prediction models. These models were applied to GWAS data, including 29,266 lung cancer cases and 56,450 controls, to assess the associations of genetically predicted gene expression levels with lung cancer risk.

**Results:**

A total of 8624 genes were successfully built for single‐tissue models, and 11,341 genes for joint‐tissue models (12,133 unique genes altogether). Among 40 genes whose expression levels were associated with the risk of lung cancer at a Bonferroni‐corrected significance level, *ZKSCAN4* was located more than 2 Mb away from the GWAS‐identified variants linked to lung cancer. Among the remaining 39 genes within 2 Mb of GWAS‐identified variants, seven genes were independent of these. Among 53 genes associated with the risk of lung cancer subtypes, 13 genes were beyond 2 Mb of GWAS‐identified variants, and four genes were independent of the GWAS‐identified variants within 2 Mb regions.

**Conclusion:**

Our TWAS identified over 50 candidate susceptibility genes for lung cancer, providing new insights into lung cancer genetics.

## Graphical abstract

A transcriptome‐wide association study identified 40 genes associated with the risk of lung cancer, leveraging gene expression and genotype data from the Genotype‐Tissue Expression (GTEx) project and summary statistics of a genome‐wide association study on lung cancer.

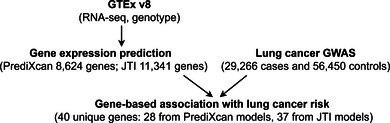

## 1 Introduction

Lung cancer (LC) is one of the most common cancers and a leading cause of cancer‐related mortality worldwide, affecting both men and women [1]. Tobacco smoking remains the primary risk factor associated with the development of LC [1]. Other risk factors include alcohol consumption, unhealthy diet, family history of lung diseases, and various environmental and occupational exposures. Genetic factors significantly influence lung cancer risk. The heritability of lung cancer estimated from family‐based studies and genome‐wide association studies (GWAS) ranges from 7% to 21% [2, 3, 4, 5]. Recent GWAS have identified approximately 80 genomic regions harboring single nucleotide polymorphisms (SNPs) associated with the risk of developing lung cancer and its specific histological subtypes [6, 7, 8, 9, 10, 11]. As a powerful hypothesis‐free approach, GWAS can uncover novel genetic variants for diseases of interest, such as LC. However, it is restricted by only studying common single genetic variants, missing heritability, difficulty in pinpointing causal genetic variants or genes, and replicating findings in different populations, and limited predictive values in disease risks or outcomes [7, 12, 13]. Specifically, the causal susceptibility genes are still unknown for the majority of the lung cancer GWAS‐identified genetic loci, although gene expression quantitative trait loci (eQTL) analyses have provided valuable insights into the potential roles of certain genes in lung cancer development [6, 7, 8, 9, 10, 11, 14]. Further research is needed to uncover the putative causal genes and elucidate their roles in lung cancer carcinogenesis.

Transcriptome‐wide association study (TWAS) is a gene‐based approach that integrates gene expression data with GWAS data to identify genes potentially involved in the development of complex traits and diseases [12, 15, 16, 17, 18, 19, 20, 21, 22]. TWAS has been applied to identify candidate susceptibility genes for a variety of complex diseases, including cancers [23, 24, 25, 26, 27, 28]. In 2020, a TWAS on lung cancer identified 36 genes for overall lung cancer risk and an additional 14 genes linked to lung cancer sub‐types [26]. That study utilized GTEx version 7 (v7) along with an in‐house gene expression dataset. However, a key limitation was the use of the Affymetrix array for gene expression profiling, which can produce low background signals and result in inaccurate expression levels for certain genes. Additionally, the study employed GTEx v7 for replication, while the updated GTEx version 8 is now available, offering improved data for TWAS analysis.

In the present study, we analyzed RNA‐Seq data from lung tissue and 48 other tissue types, encompassing sample sizes ranging from 73 to 706, to build lung‐tissue‐based and joint‐tissue‐based gene prediction models. These models were subsequently applied to lung cancer GWAS data to identify candidate genes potentially involved in lung cancer development. By leveraging these comprehensive datasets, our aim is to gain a deeper understanding of the genetic basis of lung cancer.

## 2 Materials and Methods

### 2.1 GTEx Dataset

The GTEx data v8 [29] was used to develop gene expression prediction models. For lung‐tissue‐based models, RNA‐Seq data from lung tissue and whole‐genome sequencing data from 444 participants of European ancestry were included. These subjects were uniquely selected because they had both genotype and gene expression data accessible for the study. Gene expression levels were quantified using reads per kilobase per million units derived from RNA‐Seq. We conducted data quality control and normalization processing by filtering poorly expressed genes (missing in ≥ 50% of samples and those with counts < 6 in ≥ 50% of the samples), log_2_ transforming, and quantile normalization. In addition, for lung tissue data, we conducted rank‐based inverse normal transformation for gene expression across all samples to improve normality and mitigate the impact of extreme values. To account for batch effects and other potential confounding factors, we integrated 60 factors estimated through probabilistic estimation of expression residuals (PEER) analysis [30] into the gene expression prediction model‐building process. For genotype data from whole‐genome sequencing, only variants meeting the following criteria were included for model building: (1) call rate > 98%; (2) *p*‐value > 10^−6^ for Hardy–Weinberg equilibrium test; and (3) minor allele frequency (MAF) > 0.05. Principal component analyses (PCA) were conducted using PLINK 2.0 [31].

### 2.2 Building Gene Expression Prediction Models

**FIGURE 1:**
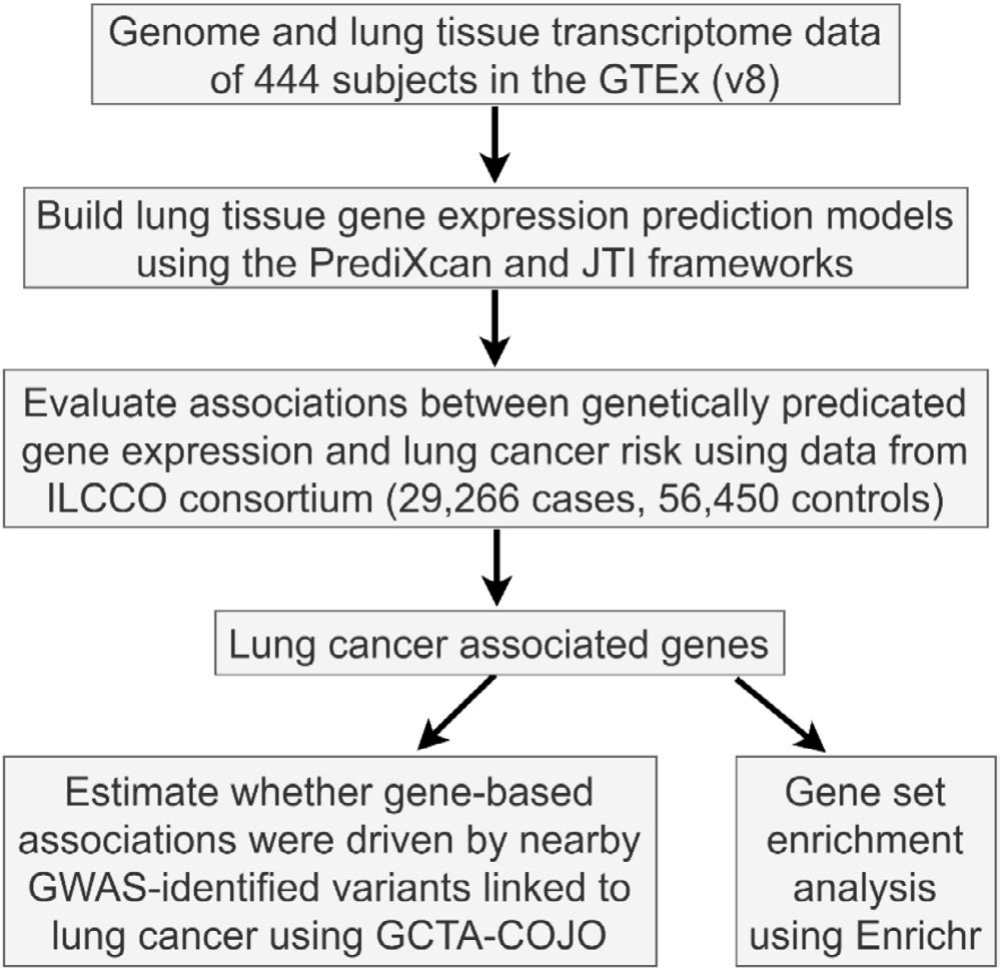
Study design flow chart.

The overall study design is presented in Figure 1. Given our hypothesis that gene expression in lung tissue plays a critical role in lung cancer, we used the PrediXcan framework to develop single‐tissue models. The normalized gene expression data from normal lung tissue was regressed on covariates, including age, sex, smoking status, the first five principal components, and 60 PEER factors, to obtain residuals. For each gene, a statistical model was built using allelic dosage data from *cis*‐acting SNPs (within 500 kb flanking each gene) to predict gene expression residuals by using the elastic net method [23, 32], with *α* = 0.5 and five‐fold cross‐validation.

In GTEx v8, gene expression data from lung tissue and 48 other types of tissue, with sample sizes ranging from 73 to 706 individuals, were also available. These data, together with the regulatory profile of the DNase I hypersensitivity sites in promoter regions for different tissues and cell types from ENCODE/Roadmap, were used to estimate similarity between lung tissue and each of the other tissues. The tissue‐pair similarity from gene expression and regulatory profile was combined using hyperparameters, which showed the best fit with the minimum tuning error via a grid search in five‐fold cross‐validation. This gene prediction framework, the joint‐tissue imputation (JTI) model, has been demonstrated as a powerful approach for TWAS and has been described in detail previously [21]. For both single‐tissue and JTI models, only genes with adequate prediction performance, defined by a five‐fold cross‐validation Pearson correlation coefficient (*r*) ≥ 0.1 and *p*‐value < 0.05 between observed and predicted expression, were considered imputable and selected for downstream analyses.

### 2.3 Lung Cancer GWAS Dataset

Summary statistics from the lung cancer GWAS by McKay et al. [6] were obtained from dbGaP (https://www.ncbi.nlm.nih.gov/gap/) under accession number phs001273.v1.p1. The GWAS data were derived from the Transdisciplinary Research in Cancer of the Lung team of the International Lung Cancer Consortium (TRICL‐ILCCO) OncoArray project, which included participants of European ancestry genotyped using the OncoArray and other Illumina genome‐wide arrays. Summary statistics were available for overall lung cancer (29,266 cases and 56,450 controls) and major histological subtypes, including lung adenocarcinoma (LUAD, 11,273 cases and 55,483 controls), lung squamous cell carcinoma (LUSC; 7426 cases and 55,627 controls), and small cell carcinoma (SCC; 2664 cases and 21,444 controls). The gene expression prediction models were applied to the lung cancer GWAS summary statistics to test the associations between predicted gene expression levels and lung cancer risk.

### 2.4 Associations Between Predicted Gene Expression and Lung Cancer Risk

In this study, the PrediXcan method [33] was used to test the associations of genetically determined gene expression with lung cancer risk based on prediction weights in the gene expression prediction models. Each prediction model was applied to the lung cancer GWAS summary statistics [6] using S‐PrediXcan [17]. The detailed methodology has been described in a previous publication [34]. The formula for this method is listed below:

$$Z_{g}=\sum_{l\in {\text{Model}}_{g}}\left(W_{\mathrm{sg}}\frac{\hat{\sigma_{s}}}{\hat{\sigma_{g}}}\frac{\hat{\beta_{s}}}{\hat{\mathrm{se}(\beta_{s}})}\right)$$

A *Z*‐score was calculated to estimate the association between the predicted gene expression and lung cancer risk. In this context, *w*_*sg*_ represents the weight of variant *s* used in predicting the expression of gene *g*. The term $\hat{\beta_{s}}$ and $\hat{\mathrm{se}(\beta_{s}})$ refer to the effect size of association and the standard error for variant *s* in lung cancer GWAS summary statistics, respectively. Additionally, $\hat{\sigma_{s}}$ and $\hat{\sigma_{g}}$ denote the estimated variances of variant *s* and the predicted expression of gene *g*, respectively. We only assessed the correlations between variants included in the prediction models for this study. The Bonferroni correction method was applied to adjust for multiple comparisons, setting a significance threshold at a *p‐value* of < 0.05 divided by the total number of gene prediction models for overall lung cancer or for each lung cancer subtype.

### 2.5 TWAS Analyses Conditional on GWAS‐Identified Lung Cancer Risk Variants

To determine whether the observed associations between predicted gene expression levels and lung cancer risk were driven by previous GWAS‐identified lung cancer risk lead variants with the strongest association, we conducted conditional analyses by adjusting for the nearest lead SNPs in the corresponding genomic regions. For each promising gene, the strongest GWAS‐identified risk SNP located within 2 megabases (Mb) flanking the transcription start or stop sites of the gene was adjusted. Each SNP, which was included in the prediction models, was tested in association with lung cancer risk by adjusting the nearest GWAS lead variants using the conditional and joint analysis (COJO) method, which accounts for linkage disequilibrium (LD) between SNPs [35]. Genotype data from 503 Europeans in the 1000 Genomes Project Phase 3 were used to estimate LD. The adjusted lung cancer association results (betas and standard errors) of these SNPs were used to rerun the S‐PrediXcan analyses to evaluate the associations of genetically predicted levels of gene expression with cancer risk conditioning on the GWAS‐identified lead variants.

### 2.6 Functional Enrichment Analyses Using Enrichr

We conducted Gene Ontology (GO), Reactome, and Kyoto Encyclopedia of Genes and Genomes (KEGG) pathway analyses for the 55 TWAS‐identified protein‐coding genes associated with lung cancer risk, using the Enrichr interactive gene knowledge discovery web server [36, 37]. Only pathways enriched with more than two genes were considered. Statistical significance was defined as a Benjamini‐Hochberg adjusted *p*‐value (i.e., *q*‐value) of < 0.05 within each database.

## 3 Results

### 3.1 Lung Tissue Gene Expression Prediction Model Building

Lung‐tissue models were successfully built for 8624 genes (7806 protein‐coding and 818 lincRNA) with a model performance of *r* ^2^ ≥ 0.01 and a *p*‐value of < 0.05 (Table S1). Joint‐tissue models were successfully built for 11,341 genes (10,062 protein‐coding and 1279 lincRNA). In total, 12,133 unique genes, including 10,768 protein‐coding and 1365 lincRNA genes, had either lung‐tissue‐based models or joint‐tissue‐based models successfully built. Among them, 7832 genes were successfully predicted for expression by both lung‐tissue and joint‐tissue‐based models. More genetic variants were used to predict gene expression in lung‐tissue models than in the joint‐tissue models, with a median (interquartile range, IQR) number of 24 (15, 37) genetic variants per gene for lung‐tissue and 11 (6, 19) variants for joint‐tissue models. Detailed information regarding the number of prediction models built according to different performance thresholds and gene types is shown in Table S1.

### 3.2 Associations of Predicted Gene Expression With Lung Cancer Risk

Among the 8624 genes whose gene expression prediction models were successfully built in lung tissue, 28 genes were identified whose predicted expression levels were associated with overall lung cancer risk at a Bonferroni‐corrected significance level (*p* ≤ 5.8 × 10^−6^). From the 11,341 joint‐tissue models, 37 genes were associated with overall lung cancer risk at a Bonferroni‐corrected significance level (*p* ≤ 4.4 × 10^−6^). In total, among the 12,133 unique genes with either lung‐tissue or joint‐tissue models, 40 unique genes were significantly associated with overall lung cancer risk. Of these, both lung‐tissue and joint‐tissue models were available for 34 genes. Among these 34 genes, 32 genes showed associations at *p* < 0.05 in both models with consistent directions. Notably, 23 genes showed significant associations with consistent association direction at a Bonferroni‐corrected significance level in both models (Figure S1; Tables 1, 2, 3).

**TABLE 1:** Thirteen unique significant genes associated with either overall or subtype specific lung cancer risk located at genomic loci at least 2 Mb away from any GWAS‐identified lung cancer risk variants.

Lung cancer	Region	Gene	Type	Lung‐tissue TWAS				Joint‐tissue TWAS			
				Gene prediction model		Association with lung cancer		Gene prediction model		Association with lung cancer	
				*R* ^2^ ^b^	No. of SNPs	*Z*‐score	*p*	*R* ^2^ ^b^	No. of SNPs	*Z*‐score	*p*
Overall	6p22.1	*ZKSCAN4*	Coding	< 0.01	5	5.02	5.19E‐07	0.01	2	5.24	1.60E‐07
LUAD	1p31.1	*FUBP1*	Coding	0.09	9	−5.44	5.18E‐08	0.09	10	−5.29	1.20E‐07
	9p13.3	*AQP3* ^a^	Coding	0.15	23	4.75	2.03E‐06	0.22	15	5.04	4.74E‐07
LUSC	6p22.1	*ZSCAN26* ^a^	Coding	0.10	30	−5.29	1.22E‐07	0.15	31	−4.86	1.17E‐06
	6p22.1	*ZSCAN9*	Coding	0.01	41	2.50	1.25E‐02	0.07	17	5.01	5.32E‐07
	6p22.1	*ZKSCAN3* ^a^	Coding	0.02	4	−4.54	5.67E‐06	0.05	14	−1.60	1.11E‐01
	6p22.2	*U91328.19*	lincRNA	0.36	50	4.58	4.68E‐06	0.37	14	4.45	8.67E‐06
	10q24.31	*BLOC1S2* ^a^	Coding	0.07	18	−4.86	1.17E‐06	0.14	42	−5.03	4.81E‐07
SCC	4q32.3	*MARCH1*	Coding	0.11	21	4.41	1.03E‐05	0.13	6	4.71	2.50E‐06
	4q32.2	*TMA16* ^a^	Coding	0.03	8	−4.52	6.10E‐06	0.05	7	−4.91	8.89E‐07
	4q32.2	*RP11‐218F10.3*	lincRNA	0.30	36	4.56	5.16E‐06	0.32	12	4.77	1.80E‐06
	6p21.1	*FRS3*	Coding	0.04	23	4.47	7.99E‐06	0.07	14	5.20	1.98E‐07
	6p22.2	*BTN2A2*	Coding	0.11	25	5.01	5.43E‐07	0.12	12	4.69	2.72E‐06

^a^ Have been reported in the previous TWAS [26].

^b^ Square of the Pearson's correlation coefficient (*r*) between the observed and the predicted expression.

TWAS results by histological subtypes were summarized in Tables 1, 4, and 5. Among the 40 genes significantly associated with overall lung cancer, five were also linked to specific subtypes: two genes (*PRPF6*, *UCKL1*) were associated with LUAD, and three genes (*HCG22, CYP21A2, IREB2*) with LUSC; all five associations were independent of nearby GWAS variants (Table 4). In addition, 47 other genes were significantly associated with lung cancer subtypes, including 15 for LUAD and 32 for LUSC.

In total, our TWAS identified 40, 17, 34, and 7 genes significantly associated with the risk of overall lung cancer, LUAD, LUSC, and SCC, respectively. These genes were summarized in Figure S2, grouped according to (1) genomic location relative to known GWAS loci, (2) dependence or independence from GWAS‐identified variants within those loci, and (3) prior identification in previous TWAS. The detailed findings for these categorized TWAS‐identified genes were provided in the sections below.

### 3.3 Thirteen Lung Cancer Risk Genes Located Far Away From GWAS‐Identified Loci

Among the 13 unique TWAS‐identified genes located more than 2 Mb away from known GWAS variants (Table 1), genetically predicted expression of *ZKSCAN4* was significantly associated with an increased risk of overall lung cancer, with *p*‐values of 5.19 × 10^−7^ and 1.60 × 10^−7^ in the lung‐tissue and joint‐tissue models, respectively.

These 13 genes were significantly associated with the risk of lung cancer subtypes. Two genes, *FUBP1* (*p* = 5.18 × 10^−8^ for lung‐tissue model, *p* = 1.20 × 10^−7^ for the joint‐tissue model) and *AQP3* (*p* = 2.03 × 10^−6^ for the lung‐tissue model, *p* = 4.74 × 10^−7^ for the joint‐tissue model), were associated with LUAD. Six genes were significantly associated with LUSC risk, including *ZKSCAN4* (*p* = 3.78 × 10^−8^ for the lung‐tissue model, *p* = 3.67 × 10^−9^ for the joint‐tissue model), *ZSCAN26* (*p* = 1.22 × 10^−7^ for the lung‐tissue model, *p* = 1.17 × 10^−6^ for the joint‐tissue model), *ZSCAN9* (*p* = 0.013 for the lung‐tissue model, *p* = 5.32 × 10^−7^ for the joint‐tissue model), *ZKSCAN3* (*p* = 5.67 × 10^−6^ for the lung‐tissue model, *p* = 0.111 for the joint‐tissue model), *U91328.19* (*p* = 4.68 × 10^−6^ for the lung‐tissue model, *p* = 8.67 × 10^−6^ for the joint‐tissue model), and *BLOC1S2* (*p* = 1.17 × 10^−6^ for the lung‐tissue model, *p* = 4.81 × 10^−7^ for the joint‐tissue model). Five genes, *MARCH1* (*p* = 1.03 × 10^−5^ for the lung‐tissue model, *p* = 2.50 × 10^−6^ for the joint‐tissue model), *TMA16* (*p* = 6.10 × 10^−6^ for the lung‐tissue model, *p* = 8.89 × 10^−7^ for the joint‐tissue model), *RP11‐218F10.3* (*p* = 5.16 × 10^−6^ for the lung‐tissue model, *p* = 1.80 × 10^−6^ for the joint‐tissue model), *FRS3* (*p* = 7.99 × 10^−6^ for the lung‐tissue model, *p* = 1.98 × 10^−7^ for the joint‐tissue model), and *BTN2A2* (*p* = 5.43 × 10^−7^ for the lung‐tissue model, *p* = 2.27 × 10^−6^ for the joint‐tissue model), were associated with SCC. The genes *ZSCAN9*, *MARCH1*, *TMA16*, and *FRS3* were exclusively identified in the joint‐tissue models and did not meet the Bonferroni correction threshold in the lung‐tissue models. Conversely, *ZKSCAN3* and *U91328.19* reached Bonferroni corrected significance exclusively in the lung‐tissue models. All the aforementioned genes demonstrated consistent directions of association in both models. The performance of the 13 genes in Table 1 across all cancer types is illustrated in Table S2 and Figure S3.

### 3.4 Eleven Lung Cancer Risk Genes Independent of GWAS‐Identified Variants

We identified 11 unique genes independent of GWAS‐identified variants for overall lung cancer or its subtypes, including six novel findings. Among the 39 significant genes located within a 2 Mb region of known GWAS‐identified genetic variants, seven remained significantly associated with lung cancer risk after adjusting for nearby GWAS‐identified variants. Of these seven genes, *IREB2*, *CHRNA5*, and *CHRNA3* exhibited strong evidence of independent associations, with adjusted *p*‐values of 4.08 × 10^−11^, 4.04 × 10^−7^, and 6.49 × 10^−8^, respectively (Table 2). These findings suggest that the associations of these three genes are independent of nearby GWAS variants. The remaining four genes (*RNF39*, *PPP1R11*, *UCKL1*, and *PRPF6*) showed moderate evidence of independent signaling, with adjusted *p*‐values of 1.73 × 10^−5^, 8.30 × 10^−5^, 1.66 × 10^−5^, and 1.23 × 10^−5^, respectively (Table 2). Three genes (*RNF39*, *UCKL1*, and *PRPF6*) have not been reported in previous TWAS, representing novel findings.

**TABLE 2:** Seven significant genes associated with overall lung cancer risk that are located at loci within 2 Mb and independent of nearby GWAS‐identified lung cancer risk variants.

Region	Gene	Type	Lead GWAS variant	Lung‐tissue TWAS					Joint‐tissue TWAS				
				*R* ^2^ ^b^	No. of SNPs	*Z*‐score	*p*	*p*_adjusted_	*R* ^2^ ^b^	No. of SNPs	*Z*‐score	*p*	*p*_adjusted_
6p22.1	*RNF39*	Coding	rs114002231	0.05	35	−5.31	1.08E‐07	6.96E‐03	0.07	22	−7.28	3.25E‐13	1.73E‐05
6p22.1	*PPP1R11* ^a^	Coding	rs114002231	0.06	94	−1.48	1.38E‐01	—	0.04	13	−6.13	9.01E‐10	8.30E‐05
15q25.1	*IREB2* ^a^	Coding	rs55781567	0.03	17	−12.84	9.83E‐38	7.37E‐03	0.02	15	−15.15	7.75E‐52	4.08E‐11
15q25.1	*CHRNA5* ^a^	Coding	rs55781567	0.09	60	−4.91	9.31E‐07	1.85E‐06	0.13	15	−9.91	3.81E‐23	4.04E‐07
15q25.1	*CHRNA3* ^a^	Coding	rs55781567	—	—	—	—	—	0.03	4	−7.21	5.41E‐13	6.49E‐08
20q13.33	*UCKL1*	Coding	rs75031349	0.03	12	−4.66	3.14E‐06	1.66E‐05	0.06	29	−4.69	2.78E‐06	3.04E‐05
20q13.33	*PRPF6*	Coding	rs75031349	0.08	14	4.82	1.43E‐06	1.23E‐05	0.09	51	3.93	8.50E‐05	—

^a^ Have been reported in the previous TWAS [26].

^b^ Square of the Pearson's correlation coefficient (*r*) between the observed and the predicted expression.

For the lung cancer subtypes, we identified seven genes associated with subtype‐specific risk that were independent of GWAS‐identified genetic variants (Table 4). This included two novel findings for LUAD and two novel findings for LUSC. Three genes (*PRPF6*, *UCKL1*, *IREB2*) were also identified in overall lung cancer. Among the seven genes, two (*PRPF6*, *UCKL1*) were independent of nearby GWAS variants for LUAD and five (*HLA‐DQB1*, *HCG22*, *CYP21A2*, *ABCF1*, *IREB2*) were independent of nearby GWAS variants for LUSC (Table 4). Notably, *PRPF6* (*p* = 1.56 × 10^−6^) exhibited significance solely in the lung‐tissue models at the Bonferroni‐corrected threshold. Conversely, *CYP21A2* (*p* = 4.52 × 10^−11^) and *IREB2* (*p* = 1.36 × 10^−21^) were exclusively identified in the joint‐tissue models at the Bonferroni‐corrected threshold.

### 3.5 Lung Cancer Risk Genes With Associations Driven by GWAS‐Identified Variants

For overall lung cancer, among the 39 significant genes located within a 2 Mb range of GWAS‐identified genetic variants, associations for 32 genes disappeared or were substantially attenuated after adjusting for the nearby variants, suggesting that these associations were driven by the GWAS‐identified lung cancer risk variants (Table 3). Of these, 13 genes are novel findings. Genes such as *Xxbac‐BPG27H4.8* (the long intergenic non‐protein coding RNA 2570, *LINC02570*), *ZFP57*, *MOG*, *ADAMTS7*, *MSH5*, and *HYKK* were significantly associated with overall lung cancer risk in the joint‐tissue models but could not be successfully predicted in the lung‐tissue models. Similarly, the genes *CLIC1*, *LINC00243*, *SFTA2*, and *CYP21A2* showed Bonferroni‐corrected significance only in the joint‐tissue models, and genes *CSNK2B* and *LY6G5B* reached Bonferroni‐corrected significance only in the lung‐tissue models. Gene *PSMA4* showed Bonferroni‐corrected significance in both models, but its joint‐tissue model reliability was limited due to Pearson's correlation coefficient *r* < 0.1.

**TABLE 3:** Thirty‐two significant genes associated with overall lung cancer risk that are located at loci within 2 Mb of and driven by nearby GWAS‐identified lung cancer risk variants.

Region	Gene	Type	Lead GWAS variant	Distance (kb)^c^	Lung‐tissue TWAS					Joint‐tissue TWAS				
					Gene prediction model		Association with lung cancer			Gene prediction model		Association with lung cancer		
					*R* ^2^ ^b^	No. of SNPs	*Z*‐score	*p*	*p*_adjusted_	*R* ^2^ ^b^	No. of SNPs	*Z*‐score	*p*	*p*_adjusted_
1p31.1	*FUBP1*	Coding	rs34517439	5.7	0.09	9	−6.10	1.07E‐09	3.02E‐01	0.09	10	−6.15	7.81E‐10	1.93E‐01
6p21.32	*HLA‐DQB1* ^a^	Coding	rs28688825 rs114002231	40.1 1199.8	0.71	114	−4.84	1.32E‐06	1.30E‐04	0.67	29	−5.21	1.88E‐07	1.53E‐01
6p21.32	*RNF5*	Coding	rs28688825 rs114002231	438.6 718.7	0.05	34	−6.18	6.36E‐10	1.10E‐01	0.08	5	−5.80	6.48E‐09	2.53E‐01
6p21.33	*FLOT1* ^a^	Coding	rs114002231 rs28688825	716.9 1876.6	0.16	30	7.05	1.81E‐12	5.00E‐01	0.19	20	6.75	1.43E‐11	1.27E‐01
6p21.33	*PPP1R18* ^a^	coding	rs114002231 rs28688825	771.7 1931.5	0.06	19	−7.41	1.26E‐13	1.47E‐01	0.06	16	−6.66	2.68E‐11	8.76E‐02
6p21.33	*DPCR1*	Coding	rs114002231 rs28688825	505.4 1665.2	0.02	19	−4.86	1.19E‐06	4.94E‐02	0.02	2	−6.98	3.01E‐12	6.64E‐03
6p21.33	*GPANK1* ^a^	Coding	rs114002231 rs28688825	201.6 953.1	0.02	17	−8.27	1.33E‐16	8.44E‐01	0.07	28	−5.18	2.23E‐07	2.60E‐01
6p21.33	*APOM* ^a^	Coding	rs114002231 rs28688825	192.8 961.2	0.04	45	−4.99	6.05E‐07	2.04E‐01	0.05	20	−9.30	1.39E‐20	6.67E‐03
6p21.33	*CCHCR1* ^a^	Coding	rs114002231 rs28688825	301.4 1461.1	0.41	62	5.82	6.00E‐09	9.79E‐01	0.41	37	5.21	1.87E‐07	4.99E‐01
6p21.33	*LINC00243*	lincRNA	rs114002231 rs28688825	629.0 1788.7	0.16	47	4.67	3.06E‐06	6.33E‐01	0.15	17	5.86	4.56E‐09	8.59E‐02
6p21.33	*C4A* ^a^	Coding	rs114002231 rs28688825	522.4 616.7	0.36	65	−7.21	5.74E‐13	9.03E‐02	0.33	63	−8.17	3.08E‐16	2.14E‐02
6p21.33	*SFTA2* ^a^	Coding	rs114002231 rs28688825	504.0 1663.8	0.19	31	3.75	1.77E‐04	—	0.28	15	4.92	8.66E‐07	4.87E‐02
6p21.33	*MSH5*	Coding	rs114002231 rs28688825	280.3 856.5	< 0.01	34	−2.25	2.42E‐02	—	0.01	3	−6.71	1.93E‐11	3.73E‐01
6p21.33	*CLIC1*	Coding	rs114002231 rs28688825	271.0 879.6	0.04	19	−2.56	1.06E‐02	—	0.01	33	−7.01	2.44E‐12	5.43E‐02
6p21.33	*HCG22*	lincRNA	rs114002231 rs28688825	399.7 1559.5	0.28	48	−4.28	1.89E‐05	—	0.27	15	−4.66	3.15E‐06	7.96E‐04
6p21.33	*CYP21A2* ^a^	Coding	rs28688825 rs114002231	577.7 578.6	0.07	37	3.20	1.39E‐03	—	0.16	50	5.14	2.74E‐07	9.99E‐03
6p21.33	*XXbac‐BPG27H4.8*	lincRNA	rs114002231 rs28688825	611.5 1771.2	—	—	—	—	—	0.08	15	4.77	1.80E‐06	3.72E‐02
6p21.33	*CSNK2B*	Coding	rs114002231 rs28688825	205.6 949.0	0.02	19	5.67	1.41E‐08	9.05E‐01	0.04	13	−0.51	6.10E‐01	—
6p21.33	*LY6G5B*	Coding	rs114002231 rs28688825	210.5 945.6	0.28	37	4.72	2.36E‐06	4.21E‐01	0.29	35	3.62	2.97E‐04	—
6p22.1	*ZNRD1* ^a^	Coding	rs114002231	1394.7	0.13	56	−5.96	2.47E‐09	1.45E‐03	0.15	23	−4.85	1.27E‐06	3.55E‐03
6p22.1	*TRIM31* ^a^	Coding	rs114002231	1346.5	0.06	31	−6.58	4.59E‐11	3.20E‐02	0.12	21	−7.70	1.31E‐14	1.25E‐03
6p22.1	*HCP5B* ^a^	lincRNA	rs114002231	1585.8	0.63	86	6.31	2.70E‐10	2.53E‐01	0.57	18	5.87	4.48E‐09	1.00E‐02
6p22.1	*ZFP57* ^a^	Coding	rs114002231	1778.5	—	—	—	—	—	0.61	34	6.69	2.17E‐11	4.21E‐03
6p22.1	*MOG*	Coding	rs114002231	1787.2	—	—	—	—	—	0.02	3	7.19	6.61E‐13	1.69E‐03
6q27	*RNASET2* ^a^	Coding	rs6920364	5.8	0.23	41	4.91	8.92E‐07	7.17E‐01	0.26	19	4.93	8.19E‐07	8.65E‐01
11q23.3	*MPZL3* ^a^	Coding	rs11607355	3.9	0.12	16	−5.04	4.56E‐07	5.99E‐01	0.11	7	−5.46	4.76E‐08	2.46E‐01
12p13.33	*RAD52* ^a^	Coding	rs7953330	22.4	0.16	17	5.82	5.95E‐09	1.55E‐01	0.28	20	5.43	5.63E‐08	3.88E‐01
15q21.1	*SECISBP2L* ^a^	Coding	rs2413932 rs66759488	44.7 1703.2	0.18	11	−5.73	9.88E‐09	3.06E‐01	0.20	12	−6.05	1.45E‐09	2.27E‐01
15q25.1	*ADAMTS7*	Coding	rs55781567	193.6	—	—	—	—	—	0.01	5	5.34	9.21E‐08	3.60E‐02
15q25.1	*HYKK* ^a^	Coding	rs55781567	28.3	—	—	—	—	—	0.01	19	−6.33	2.45E‐10	3.47E‐01
15q25.1	*PSMA4* ^a^	Coding	rs55781567	13.2	0.01	23	16.19	6.43E‐59	6.96E‐02	< 0.01	7	8.32	8.98E‐17	—
19q13.	*CYP2A6*	Coding	rs56113850	3.2	0.02	9	8.40	4.58E‐17	4.69E‐03	0.05	12	8.80	1.42E‐18	5.27E‐03

^a^ Have been reported in the previous TWAS [26].

^b^ Square of the Pearson's correlation coefficient (*r*) between the observed and the predicted expression.

^c^ Distance between the lead SNP and gene body.

**TABLE 4:** Seven genes associated with subtype‐specific lung cancer risk that are located at loci within 2 Mb and independent of nearby GWAS‐identified risk variants.

Lung cancer	Region	Gene	Type	Lead GWAS variant	Distance (kb)^c^	Lung‐tissue TWAS					Joint‐tissue TWAS				
						Gene prediction model		Association with lung cancer			Gene prediction model		Association with lung cancer		
						*R* ^2^ ^b^	No. of SNPs	*Z*‐score	*p*	*p*_adjusted_	*R* ^2^ ^b^	No. of SNPs	*Z*‐score	*p*	*p*_adjusted_
LUAD	20q13.33	*PRPF6*	Coding	rs75031349	298.4	0.08	14	4.80	1.56E‐06	1.23E‐05	0.09	51	4.56	5.16E‐06	—
	20q13.33	*UCKL1*	Coding	rs75031349	257.1	0.03	12	−5.23	1.74E‐07	1.66E‐05	0.06	29	−4.98	6.45E‐07	4.63E‐05
LUSC	6p21.32	*HLA‐DQB1* ^a^	Coding	rs114002231	1199.8	0.71	114	−4.99	6.13E‐07	1.30E‐04	0.67	29	−5.34	9.38E‐08	6.66E‐05
	6p21.33	*CYP21A2* ^a^	Coding	rs114002231	578.6	0.07	37	4.33	1.47E‐05	—	0.16	50	6.59	4.52E‐11	5.42E‐05
	6p21.33	*ABCF1*	Coding	rs114002231	862.4	0.01	24	5.14	2.75E‐07	6.83E‐05	0.03	13	4.79	1.68E‐06	1.06E‐04
	6p21.33	*HCG22*	lincRNA	rs114002231	399.7	0.28	48	−4.59	4.50E‐06	5.06E‐05	0.27	15	−5.28	1.32E‐07	2.42E‐05
	15q25.1	*IREB2* ^a^	Coding	rs55781567	64.2	0.03	17	−4.44	8.89E‐06	—	0.02	15	−9.55	1.36E‐21	8.26E‐07

^a^ Have been reported in the previous TWAS [26].

^b^ Square of the Pearson's correlation coefficient (*r*) between the observed and the predicted expression.

^c^ Distance between the lead SNP and gene body.

**TABLE 5:** Thirty‐four unique genes associated with lung cancer subtype risk that are located at loci within 2 Mb of and driven by nearby GWAS‐identified risk variants.

Lung cancer subtype	Region	Gene	Type	Lead GWAS variant	Distance (kb)^c^	Lung‐tissue TWAS					Joint‐tissue TWAS				
						Gene prediction model		Association with lung cancer			Gene prediction model		Association with lung cancer		
						*R* ^2^ ^b^	No. of SNPs	*Z*‐score	*p*	*p*_adjusted_	*R* ^2^ ^b^	No. of SNPs	*Z*‐score	*p*	*p*_adjusted_
LUAD	3q28	*TP63* ^a^	Coding	rs36108040	13.4	0.02	31	−5.39	7.08E‐08	4.59E‐01	0.04	9	−5.08	3.79E‐07	8.51E‐01
	3q28	*TPRG1*	Coding	rs36108040	292.7	0.03	54	−4.68	2.93E‐06	3.46E‐03	—	—	—	—	—
	6q22.1	*DCBLD1* ^a^	Coding	rs9374662	2.8	0.25	49	−4.90	9.64E‐07	5.65E‐01	0.30	22	−4.72	2.37E‐06	6.72E‐01
	8p12	*NRG1* ^a^	Coding	rs7823498	221.9	0.06	39	−6.15	7.67E‐10	2.64E‐02	0.06	3	−4.96	7.17E‐07	2.06E‐01
	10q24.33	*STN1*	Coding	rs7902587	16.3	< 0.01	14	−5.08	3.81E‐07	—	0.02	2	−6.53	6.76E‐11	1.27E‐01
	11q23.3	*MPZL3* ^a^	Coding	rs11607355	3.9	0.12	16	−5.41	6.33E‐08	5.99E‐01	0.11	7	−6.08	1.22E‐09	1.42E‐01
	15q21.1	*SECISBP2L* ^a^	Coding	rs2413932	44.7	0.18	11	−7.96	1.78E‐15	3.06E‐01	0.20	12	−8.35	6.87E‐17	1.49E‐01
	15q21.2	*FAM227B* ^a^	Coding	rs2413932	235.7	0.11	28	4.02	5.76E‐05	—	0.11	14	5.52	3.36E‐08	4.47E‐01
	15q25.1	*PSMA4* ^a^	Coding	rs55781567	13.2	0.01	23	11.81	3.50E‐32	6.96E‐02	< 0.01	7	6.26	3.88E‐10	—
	15q25.1	*IREB2* ^a^	Coding	rs55781567	64.2	0.03	17	−7.10	1.21E‐12	7.37E‐03	0.02	15	−9.03	1.65E‐19	1.12E‐03
	15q25.1	*CHRNA5* ^a^	Coding	rs55781567	0.1	0.09	60	−3.11	1.90E‐03	—	0.13	15	−6.84	7.88E‐12	3.26E‐03
	15q25.1	*CHRNA3* ^a^	Coding	rs55781567	27.4	—	—	—	—	—	0.03	4	−5.35	9.00E‐08	1.43E‐03
	19q13.2	*CYP2A6*	Coding	rs56113850	3.2	0.02	9	5.67	1.47E‐08	4.69E‐03	0.05	12	6.79	1.15E‐11	6.12E‐03
LUSC	6p21.32	*RNF5*	Coding	rs114002231	718.7	0.05	34	−5.91	3.38E‐09	1.10E‐01	0.08	5	−5.59	2.31E‐08	1.51E‐01
	6p21.33	*FLOT1* ^a^	Coding	rs114002231	716.9	0.16	30	6.20	5.71E‐10	5.00E‐01	0.19	20	5.94	2.83E‐09	4.24E‐01
	6p21.33	*PPP1R18* ^a^	Coding	rs114002231	771.7	0.06	19	−6.30	2.91E‐10	1.47E‐01	0.06	16	−5.60	2.08E‐08	4.53E‐01
	6p21.33	*GPANK1* ^a^	Coding	rs114002231	201.6	0.02	17	−7.33	2.23E‐13	8.44E‐01	0.07	28	−4.80	1.58E‐06	3.63E‐01
	6p21.33	*CCHCR1* ^a^	Coding	rs114002231	301.4	0.41	62	4.77	1.81E‐06	9.79E‐01	0.41	37	4.07	4.80E‐05	—
	6p21.33	*DDAH2* ^a^	Coding	rs114002231	267.4	0.04	11	−1.88	6.02E‐02	—	0.06	10	−4.94	8.01E‐07	9.04E‐03
	6p21.33	*C4A* ^a^	Coding	rs114002231	522.4	0.36	65	−7.39	1.45E‐13	9.03E‐02	0.33	63	−7.51	6.09E‐14	1.52E‐01
	6p21.33	*DPCR1*	Coding	rs114002231	505.4	0.02	19	−4.25	2.18E‐05	—	0.02	2	−5.83	5.40E‐09	1.73E‐01
	6p21.33	*NELFE*	Coding	rs114002231	492.5	0.03	32	−5.00	5.86E‐07	1.20E‐01	0.04	20	−0.59	5.54E‐01	—
	6p21.33	*MSH5*	Coding	rs114002231	280.3	< 0.01	34	−1.69	9.07E‐02	—	0.01	3	−6.55	5.58E‐11	3.22E‐01
	6p21.33	*CSNK2B*	Coding	rs114002231	205.6	0.02	19	5.24	1.57E‐07	9.05E‐01	0.04	13	−1.05	2.94E‐01	—
	6p21.33	*APOM* ^a^	Coding	rs114002231	192.8	0.04	45	−5.42	5.81E‐08	2.04E‐01	0.05	20	−8.50	1.96E‐17	8.64E‐02
	6p21.33	*CLIC1*	Coding	rs114002231	271.0	0.04	19	−2.54	1.11E‐02	—	0.01	33	−6.80	1.01E‐11	2.70E‐02
	6p21.33	*LINC00243*	lincRNA	rs114002231	629.0	0.16	47	5.04	4.63E‐07	6.33E‐01	0.15	17	7.14	9.04E‐13	3.67E‐03
	6p22.1	*TRIM31* ^a^	Coding	rs114002231	1346.5	0.06	31	−5.60	2.19E‐08	3.20E‐02	0.12	21	−6.65	2.87E‐11	2.78E‐02
	6p22.1	*HCP5B* ^a^	lincRNA	rs114002231	1585.8	0.63	86	5.07	4.02E‐07	2.53E‐01	0.57	18	4.74	2.16E‐06	1.42E‐01
	6p22.1	*ZFP57* ^a^	Coding	rs114002231	1778.5	—	—	—	—	—	0.61	34	4.98	6.23E‐07	2.71E‐01
	6p22.1	*RNF39*	Coding	rs114002231	1383.7	0.05	35	−4.71	2.54E‐06	6.96E‐03	0.07	22	−5.88	4.09E‐09	3.88E‐03
	6p22.1	*MOG*	Coding	rs114002231	1787.2	—	—	—	—	—	0.02	3	5.03	4.87E‐07	3.42E‐01
	6p22.1	*OR2H2*	Coding	rs114002231	1870.6	—	—	—	—	—	0.02	10	4.69	2.69E‐06	4.91E‐03
	12p13.33	*RAD52* ^a^	Coding	rs7953330	22.4	0.16	17	6.50	8.22E‐11	1.55E‐01	0.28	20	6.12	9.54E‐10	1.73E‐01
	15q25.1	*PSMA4* ^a^	Coding	rs55781567	13.2	0.01	23	9.55	1.28E‐21	6.96E‐02	0.01	7	3.91	9.37E‐05	—
	19q13.2	*CYP2A6*	Coding	rs56113850	3.2	0.02	9	6.86	6.93E‐12	4.69E‐03	0.05	12	5.85	4.96E‐09	1.67E‐01
SCC	15q25.1	*PSMA4* ^a^	Coding	rs55781567	13.2	0.01	23	5.93	3.10E‐09	6.96E‐02	0.01	7	3.16	1.60E‐03	—
	15q25.1	*IREB2* ^a^	Coding	rs55781567	64.2	0.03	17	−6.01	1.90E‐09	7.37E‐03	0.02	15	−7.18	7.14E‐13	1.10E‐04

^a^ Have been reported in the previous TWAS [26].

^b^ Square of the Pearson's correlation coefficient (*r*) between the observed and the predicted expression.

^c^ Distance between the lead SNP and gene body.

For lung cancer subtypes, we found 34 unique genes with associations driven by GWAS‐identified variants (Table 5), including 13, 23, and 2 genes driven by GWAS variants for LUAD, LUSC, and SCC, respectively. Among them, 13 unique genes have not been reported before, with three genes specific to LUAD and 11 to LUSC. Genes *TPRG1* and *PSMA4* encountered challenges in joint‐tissue model construction, while *STN1*, *MSH5*, *ZFP57, MOG*, *OR2H2*, and *CHRNA3* genes were not reliably modeled in the lung‐tissue prediction approach. Notably, genes *FAM227B*, *DDAH2*, *DPCR1*, *CLIC1*, and *CHRNA5* demonstrated significance exclusively at the Bonferroni‐corrected threshold in the joint‐tissue models, while *CCHCR1*, *NELFE*, and *CSNK2B* reached Bonferroni‐corrected significance only in lung‐tissue models.

### 3.6 Pathways Enriched With Lung Cancer TWAS Genes

Enrichr analysis showed that the 55 LC susceptibility protein‐coding genes from our TWAS were nominally enriched in five GO pathways, one KEGG pathway, and five Reactome pathways (*p* < 0.05); however, none of these reached statistical significance after multiple testing correction (all *q*‐values > 0.05, Table S3). Notably, *CHRNA3* and *CHRNA5* were annotated in four nicotine‐related Reactome pathways and the GO pathway for cholinergic synaptic transmission.

## 4 Discussion

In this study, we integrated genomic and transcriptomic data to evaluate the associations between genetically predicted gene expression levels and lung cancer risk. We identified 13 unique genes located 2 Mb away from GWAS‐identified risk variants, including one gene (*ZKSCAN4*) for overall lung cancer, two genes (*FUBP1* and *AQP3*) for LUAD, six genes (*ZSCAN26*, *ZSCAN9*, *ZKSCAN4*, *ZKSCAN3*, *U91328.19*, *BLOC1S2*) for LUSC, and five (*MARCH1*, *TMA16*, *RP11‐218F10.3*, *FRS3*, *BTN2A2*) for SCC. Additionally, 39 unique genes were located within 2 Mb of GWAS variants associated with overall lung cancer, with seven genes (*RNF39*, *PPP1R11*, *IREB2*, *CHRNA5*, *CHRNA3*, *UCKL1*, *PRPF6*) independent of these variants. For lung cancer subtypes, we found seven genes independent of nearby GWAS variants, including *PRPF6* and *UCKL1* for LUAD, and *HLA‐DQB1*, *CYP21A2*, *ABCF1*, *HCG22*, and *IREB2* for LUSC. These findings provide novel insights into the genetic architecture of lung cancer and its subtypes.

The *ZKSCAN4* gene encodes a zinc finger protein containing Krüppel‐associated box (KRAB) and SCAN domains 4. Through its KRAB transcriptional repressor motif, ZKSCAN4 is involved in the regulation of gene transcription [38]. A related gene, the retinoblastoma (RB)‐associated KRAB zinc finger (*RBANK*), has been reported to be upregulated in non‐small cell lung cancer (NSCLC) [39]. Notably, a previous TWAS identified a negative association between *ZKSCAN3* (which is located 90 kb from *ZKSCAN4* in the MHC region) levels and lung cancer risk, but this TWAS did not detect *ZKSCAN4* using either array‐based gene expression data or GTEx version 7 for prediction model building [26]. In addition to *ZKSCAN4*, two other zinc finger SCAN genes (i.e., *ZSCAN9* and *ZSCAN26*) were also novel candidates associated with LUSC risk (Table 1). Furthermore, a GWAS of familial lung cancer identified a suggestive SNP rs116043036 (*p* = 5 × 10^−7^), located between *ZSCAN4* and *ZSCAN9* [40]. Collectively, these findings suggest a potential role for zinc finger genes in the MHC region in lung cancer development, though further research is needed to confirm this hypothesis.

The gene *FUBP1* encodes a single‐stranded DNA‐binding protein that binds to multiple DNA elements. We found that elevated expression of *FUBP1* was associated with a decreased risk of lung adenocarcinoma, consistent with prior fine‐mapping and eQTL colocalization analyses of GWAS lead SNPs in two cross‐ethnic GWASs [8, 9]. However, overexpression of *FUBP1* has also been linked to DNA damage [8] and the promotion of tumor cell proliferation and motility in NSCLC [41]. These findings underscore the complexity of the relationship between genetic risk alleles, *FUBP1* gene expression, and lung cancer risk, warranting further mechanistic investigation.

The *FRS3* encodes substrate 3 of the fibroblast growth factor receptor (FGFR), a protein known to play a role in the development and progression of lung cancer [42]. Meanwhile, the gene *BTN2A2* encodes butyrophilin subfamily 2 member A2, which is involved in innate immune pathways and class I MHC‐mediated antigen processing and presentation. *BTN2A2* may inhibit T‐cell proliferation in mice [43]. Since T‐cells are crucial for immunity, they might contribute to lung cancer defense mechanisms. The *MARCH1* gene, previously implicated in ovarian cancer development via the NF‐kB pathway [44], may also influence lung tumor progression through this same mechanism [45].

In this study, our TWAS identified genetic associations between elevated expression of *FRS3*, *BTN2A2*, and *RP11‐218F10*.3 (a long intergenic non‐coding RNA gene) and an increased risk of small cell carcinoma. However, it is important to note that these findings provide only genetic evidence, and additional experimental validation is needed to confirm these associations and elucidate their biological mechanisms. Moreover, given the relatively small number of SCC cases in our GWAS dataset (*n* = 2,664), there is a possibility that some of these associations could be false positives. Future studies should aim to replicate these results in larger cohorts and investigate the functional roles of these genes in SCC and lung cancer in general.

In 2020, a lung cancer TWAS study utilized GTEx version 7 data [26]. In comparison, our study leveraged GTEx v8, which includes a larger number of lung samples, enhancing the robustness of gene expression models. Furthermore, while the 2020 study exclusively employed lung‐tissue‐based models, our study incorporated both lung‐tissue models and multi‐tissue models, providing a broader analytical perspective. In 2021, another lung cancer TWAS study focused on the Chinese population [46]. By contrast, our study centers on individuals of European ancestry. In 2022, a TWAS encompassing multiple cancers, including lung cancer, was conducted using GTEx v8 among Europeans [47]. This study employed a novel transcription factor susceptibility approach, which differs from the methodology applied in our research.

Our study, consistent with previous lung cancer TWAS findings [26], identified a total of 55 unique genes associated with susceptibility to lung cancer or its histological subtypes. Additionally, we found that the associations of 32 (17 reported) genes with overall LC, 13 (10 reported) genes with LUAD, 23 (10 reported) genes with LUSC, and two (both reported) genes with SCC were driven by nearby GWAS‐identified variants. However, it is important to acknowledge that the genes identified in our TWAS should be viewed as prioritized or ranked candidate causal genes at specific loci, rather than definitive causal genes. This is because TWAS is unable to distinguish between causal relationships and pleiotropy, particularly when multiple TWAS‐identified genes at a single locus may be co‐regulated or share an eQTL [26, 48, 49]. To establish causal associations between the expression levels of identified genes and lung cancer risk, further investigations are required. Potential approaches for these validations include Mendelian randomization [21, 50], TWAS of multi‐ancestry populations [51], and TWAS that integrates additional omics data such as proteomics, metabolomics, or epigenomics [47, 52, 53] or analysis accounting for pleiotropic effects and linkage disequilibrium of tested SNPs [49]. These additional analyses will be critical for confirming and expanding our findings, as well as for improving understanding of the complex genetic and molecular mechanisms underlying lung cancer susceptibility.

In summary, our large‐scale TWAS identified 40 genes with genetically determined expression levels associated with overall lung cancer risk, including 17 novel genes. Additionally, we identified 53 genes associated with the risk of lung cancer subtypes, 27 of which had not been previously reported. Further research, particularly in vitro functional studies, is needed to validate the biological roles of these genes, which may offer new insights into the genetics and etiology of lung cancer.

Supporting Information can be found at *Cancer Medicine* online.

## Author Contributions

**Tianying Zhao:** methodology; formal analysis; writing – original draft. **Jiajun Shi:** methodology; formal analysis; writing – original draft. **Yaohua Yang:** methodology; formal analysis; writing – review and editing. **Dan Zhou:** methodology; software; writing – review and editing. **Jie Ping:** writing – review and editing. **Shuai Xu:** formal analysis; writing – review and editing. **Lili Xu:** writing – review and editing. **Jie Wu:** writing – review and editing. **Xiao‐Ou Shu:** writing – review and editing. **Ran Tao:** writing – review and editing. **Bingshan Li:** writing – review and editing. **Wei Zheng:** writing – review and editing. **Jirong Long:** writing – review and editing; conceptualization; supervision; funding acquisition. **Qiuyin Cai:** conceptualization; supervision; funding acquisition; writing – review and editing.

## Disclosure

Code Availability: Publicly available software and packages were used throughout this study according to each developer's instructions. Researchers are encouraged to use scripts developed and maintained by our co‐author at https://github.com/gamazonlab/MR‐JTI.

## Ethics Statement

Deidentified data used in this study were derived from published genome‐wide association studies or GTEx that followed the relevant institutional review boards and participant consent procedures. The Institutional Review Boards of Vanderbilt University approved the study's protocol (IRB #202101).

## Conflicts of Interest

The authors declare no conflicts of interest.

## Supporting information


**Table S1:** Number of genes with model building performance *p* < 0.05.
**Table S2:** Performance of 13 genes located at genomic loci at least 2 Mb away from any GWAS‐identified lung cancer risk variants in all types.
**Table S3:** Enrichr‐identified nominally enriched biological pathways with the 55 candidate risk genes for lung cancer.
**Figure S1:** TWAS results for overall lung cancer.
**Figure S2:** Bar plot of TWAS‐identified genes significantly associated with risk for lung cancer overall and histological subtypes.
**Figure S3:** Performance of 13 genes located at genomic loci at least 2 Mb away from any GWAS‐identified lung cancer risk variants in all lung cancer types.

## Data Availability

The publicly available genotype and gene expression data of GTEx participants used in this study are available in the dbGaP under accession code phs000424.v8.p2. The JTI GTEx models with lung tissue as the target are available for download from Zenodo (https://doi.org/10.5281/zenodo.3842289). The lung cancer GWAS summary statistics were obtained from dbGaP under accession code phs001273.v1.p1.
