# Supplementary material for: A Transcriptome‐Wide Association Study Identifies Candidate Susceptibility Loci and Genes for Lung Cancer Risk
Source: Cancer Med. 2025 Oct 16;14(20):e71301. doi: 10.1002/cam4.71301 (PMC12529646; doi:10.1002/cam4.71301)

**Supplement to “A transcriptome-wide association study identifies candidate susceptibility loci and genes for lung cancer risk”**

Tianying Zhao, Jiajun Shi, Yaohua Yang, Dan Zhou, Jie Ping, Shuai Xu, Xiao-Ou Shu, Ran Tao, Bingshan Li, Wei Zheng, Jirong Long, Qiuyin Cai

**Contents of supplemental material**

**Supplementary Table 1.** Number of genes with model building performance *p* < 0.05.

**Supplementary Table 2.** Performance of thirteen genes located at genomic loci at least 2 Mb away from any GWAS-identified lung cancer risk variants in all types.

**Supplementary Table 3**. Enrichr-identified nominally enriched biological pathways with the 55 candidate risk genes for lung cancer.

**Supplemental Figure 1.** TWAS results for overall lung cancer.

**Supplementary Figure 2**. Bar plot of TWAS-identified genes significantly associated with risk for lung cancer overall and histological subtypes.

**Supplemental Figure 3.** Performance of thirteen genes located at genomic loci at least 2 Mb away from any GWAS-identified lung cancer risk variants in all lung cancer types.

| **Supplementary Table 1.** Number of genes with model building performance *p* < 0.05. | | | | | | |
| --- | --- | --- | --- | --- | --- | --- |
| **Model** | **Prediction performance (R^2^)** | **Protein** | **LincRNAs** | **Total** | **No. of variants per gene** ^†^ | |
|  |  |  |  |  | **Median (IQR)** | **Range** |
| PrediXcan | ≥0.01 | 7,806 | 818 | 8,624 | 24 (15, 37) | 1, 228 |
|  | ≥0.04 | 4,604 | 539 | 5,143 | 28 (18, 42) | 1, 228 |
|  | ≥0.09 | 2,710 | 353 | 3,063 | 31 (21, 47) | 1, 228 |
|  | ≥0.16 | 1,547 | 221 | 1,768 | 35 (23, 50) | 1, 228 |
| JTI | ≥0.01 | 10,062 | 1,279 | 11,341 | 11 (6, 19) | 1, 108 |
|  | ≥0.04 | 5,979 | 785 | 6,764 | 14 (8, 22) | 1, 108 |
|  | ≥0.09 | 3,340 | 490 | 3,830 | 16 (10, 25) | 1, 108 |
|  | ≥0.16 | 1,836 | 286 | 2,122 | 18 (11, 28) | 1, 108 |
| ^†^ Number of variants used in the prediction models to predict each gene. IQR: interquartile range; JTI: joint-tissue imputation; R^2^: square of the Pearson’s correlation coefficient (r) between the observed and the predicted expression. | | | | | | |

| **Supplementary Table 2.** Performance of thirteen genes located at genomic loci at least 2 Mb away from any GWAS-identified lung cancer risk variants in all types. | | | | | | | | |
| --- | --- | --- | --- | --- | --- | --- | --- | --- |
| Lung cancer |  |  |  | Lung-tissue PrediXcan | |  | JTI | |
|  | Region | Gene | Gene type | Z-score | *P*-value |  | Z-score | *P*-value |
| Overall | 1p31.1 | *FUBP1* | coding | -6.10 | **1.07E-09** |  | -6.15 | **7.81E-10** |
|  | 4q32.3 | *MARCH1* | coding | 3.16 | **1.60E-03** |  | 3.73 | **1.89E-04** |
|  | 4q32.2 | *TMA16* | coding | -3.88 | **1.05E-04** |  | -3.87 | **1.11E-04** |
|  | 4q32.2 | *RP11-218F10.3* | lincRNA | 3.58 | **3.41E-04** |  | 3.90 | **9.59E-05** |
|  | 6p21.1 | *FRS3* | coding | 1.37 | 0.17 |  | 1.99 | **0.05** |
|  | 6p22.1 | *ZSCAN9* | coding | 1.80 | 0.07 |  | 2.90 | **3.69E-03** |
|  | 6p22.1 | *ZKSCAN4* | coding | 5.02 | **5.19E-07** |  | 5.24 | **1.60E-07** |
|  | 6p22.1 | *ZKSCAN3* | coding | -3.11 | **1.86E-03** |  | 0.29 | 0.77 |
|  | 6p22.1 | *ZSCAN26* | coding | -3.79 | **1.52E-04** |  | -3.10 | **1.91E-03** |
|  | 6p22.2 | *BTN2A2* | coding | 3.75 | **1.74E-04** |  | 3.08 | **2.06E-03** |
|  | 6p22.2 | *U91328.19* | lincRNA | 3.05 | **2.32E-03** |  | 3.00 | **2.74E-03** |
|  | 9p13.3 | *AQP3* | coding | 2.50 | **0.01** |  | 2.92 | **3.50E-03** |
|  | 10q24.31 | *BLOC1S2* | coding | -3.74 | **1.84E-04** |  | -3.61 | **3.11E-04** |
| LUAD | 1p31.1 | *FUBP1* | coding | -5.44 | **5.18E-08** |  | -5.29 | **1.20E-07** |
|  | 4q32.3 | *MARCH1* | coding | 2.20 | **0.03** |  | 2.27 | **0.02** |
|  | 4q32.2 | *TMA16* | coding | -2.04 | **0.04** |  | -2.43 | **0.02** |
|  | 4q32.2 | *RP11-218F10.3* | lincRNA | 2.34 | **0.02** |  | 2.38 | **0.02** |
|  | 6p21.1 | *FRS3* | coding | -0.97 | 0.33 |  | -0.59 | 0.56 |
|  | 6p22.1 | *ZSCAN9* | coding | 0.52 | 0.60 |  | 1.52 | 0.13 |
|  | 6p22.1 | *ZKSCAN4* | coding | 1.20 | 0.23 |  | 1.30 | 0.19 |
|  | 6p22.1 | *ZKSCAN3* | coding | 0.24 | 0.81 |  | 0.91 | 0.36 |
|  | 6p22.1 | *ZSCAN26* | coding | -0.15 | 0.88 |  | 0.08 | 0.94 |
|  | 6p22.2 | *BTN2A2* | coding | 2.74 | **0.01** |  | 2.23 | **0.03** |
|  | 6p22.2 | *U91328.19* | lincRNA | 1.79 | 0.07 |  | 1.75 | 0.08 |
|  | 9p13.3 | *AQP3* | coding | 4.75 | **2.03E-06** |  | 5.04 | **4.74E-07** |
|  | 10q24.31 | *BLOC1S2* | coding | -2.40 | **0.02** |  | -2.09 | **0.04** |
| LUSC | 1p31.1 | *FUBP1* | coding | -3.76 | **1.72E-04** |  | -3.78 | **1.59E-04** |
|  | 4q32.3 | *MARCH1* | coding | 1.84 | **0.07** |  | 2.33 | **0.02** |
|  | 4q32.2 | *TMA16* | coding | -2.67 | **0.01** |  | -2.47 | **0.01** |
|  | 4q32.2 | *RP11-218F10.3* | lincRNA | 2.01 | **0.04** |  | 2.46 | **0.01** |
|  | 6p21.1 | *FRS3* | coding | 0.51 | 0.61 |  | 1.35 | 0.18 |
|  | 6p22.1 | *ZSCAN9* | coding | 2.50 | **0.01** |  | 5.01 | **5.32E-07** |
|  | 6p22.1 | *ZKSCAN4* | coding | 5.50 | **3.78E-08** |  | 5.90 | **3.67E-09** |
|  | 6p22.1 | *ZKSCAN3* | coding | -4.54 | **5.67E-06** |  | -1.60 | 0.11 |
|  | 6p22.1 | *ZSCAN26* | coding | -5.29 | **1.22E-07** |  | -4.86 | **1.17E-06** |
|  | 6p22.2 | *BTN2A2* | coding | 1.22 | 0.22 |  | 0.69 | 0.49 |
|  | 6p22.2 | *U91328.19* | lincRNA | 4.58 | **4.68E-06** |  | 4.45 | **8.67E-06** |
|  | 9p13.3 | *AQP3* | coding | -1.30 | 0.19 |  | -1.09 | 0.27 |
|  | 10q24.31 | *BLOC1S2* | coding | -4.86 | **1.17E-06** |  | -5.03 | **4.81E-07** |
| SCC | 1p31.1 | *FUBP1* | coding | -2.68 | **0.01** |  | -2.63 | **0.01** |
|  | 4q32.3 | *MARCH1* | coding | 4.41 | **1.03E-05** |  | 4.71 | **2.50E-06** |
|  | 4q32.2 | *TMA16* | coding | -4.52 | **6.10E-06** |  | -4.91 | **8.89E-07** |
|  | 4q32.2 | *RP11-218F10.3* | lincRNA | 4.56 | **5.16E-06** |  | 4.77 | **1.80E-06** |
|  | 6p21.1 | *FRS3* | coding | 4.47 | **7.99E-06** |  | 5.20 | **1.98E-07** |
|  | 6p22.1 | *ZSCAN9* | coding | 1.44 | 0.15 |  | 1.35 | 0.18 |
|  | 6p22.1 | *ZKSCAN4* | coding | 2.89 | **3.87E-03** |  | 3.20 | **1.39E-03** |
|  | 6p22.1 | *ZKSCAN3* | coding | -1.77 | 0.08 |  | 0.13 | 0.90 |
|  | 6p22.1 | *ZSCAN26* | coding | -1.67 | 0.09 |  | -1.86 | 0.06 |
|  | 6p22.2 | *BTN2A2* | coding | 5.01 | **5.43E-07** |  | 4.69 | **2.72E-06** |
|  | 6p22.2 | *U91328.19* | lincRNA | 2.66 | **0.01** |  | 2.37 | **0.02** |
|  | 9p13.3 | *AQP3* | coding | -1.22 | 0.22 |  | -1.24 | 0.21 |
|  | 10q24.31 | *BLOC1S2* | coding | -0.06 | 0.95 |  | 0.32 | 0.75 |
| Boldness indicates the statistical significance level of *p* <0.05 | | | | | | | | |

| **Supplementary Table 3**. Enrichr-identified nominally enriched biological pathways with the 55 candidate risk genes for lung cancer. | | | | |
| --- | --- | --- | --- | --- |
| Database | Term | *p*-value | *q*-value^†^ | Genes |
| GO_Biological_Process_2021 | |  |  |  |
|  | activation of transmembrane receptor protein tyrosine kinase activity (GO:0007171) | 1.11E-04 | 0.054 | *CHRNA3*;*NRG1* |
|  | regulation of cytokine production (GO:0001817) | 7.70E-04 | 0.149 | *MOG*;*BTN2A2*;*PPP1R11*;*FLOT1* |
|  | T cell receptor signaling pathway (GO:0050852) | 9.35E-04 | 0.149 | *PSMA4*;*MOG*;*BTN2A2*;*HLA-DQB1* |
|  | synaptic transmission, cholinergic (GO:0007271) | 1.23E-03 | 0.149 | *CHRNA3;CHRNA5* |
|  | antigen receptor-mediated signaling pathway (GO:0050851) | 1.67E-03 | 0.162 | *PSMA4;MOG;BTN2A2;HLA-DQB1* |
| KEGG_2021_Human | |  |  |  |
|  | Staphylococcus aureus infection | 0.028 | 0.401 | *C4A*;*HLA-DQB1* |
| Reactome_2022 | |  |  |  |
|  | Highly Calcium Permeable Nicotinic Acetylcholine Receptors R-HSA-629597 | 2.64E-04 | 0.052 | *CHRNA3*;*CHRNA5* |
|  | Highly Calcium Permeable Postsynaptic Nicotinic Acetylcholine Receptors R-HSA-629594 | 4.02E-04 | 0.052 | *CHRNA3*;*CHRNA5* |
|  | Presynaptic Nicotinic Acetylcholine Receptors R-HSA-622323 | 4.82E-04 | 0.052 | *CHRNA3*;*CHRNA5* |
|  | Acetylcholine Binding And Downstream Events R-HSA-181431 | 6.62E-04 | 0.054 | *CHRNA3*;*CHRNA5* |
|  | ABC-family Proteins Mediated Transport R-HSA-382556 | 2.79E-03 | 0.181 | *PSMA4*;*RNF5*;*ABCF1* |
| † *q*-values are adjusted *p*-values using the Benjamini-Hockberg method. | | | | |

**Supplementary Figure 1**. TWAS results for overall lung cancer. Manhattan plots for PrediXcan (top) and JTI (bottom) models are illustrated to show similarities and differences between these two TWAS approaches. Each red point represents a protein-coding or lincRNA gene significantly associated with overall lung cancer risk (dashed lines indicate Bonferroni significance thresholds, with *p*-values of 5.8×10^-6^ for PrediXcan and 4.4×10^-6^ for JTI, respectively). X axes indicate genomic positions and Y axes indicate *p*-values for gene expression-lung cancer risk associations in -log10 scale.


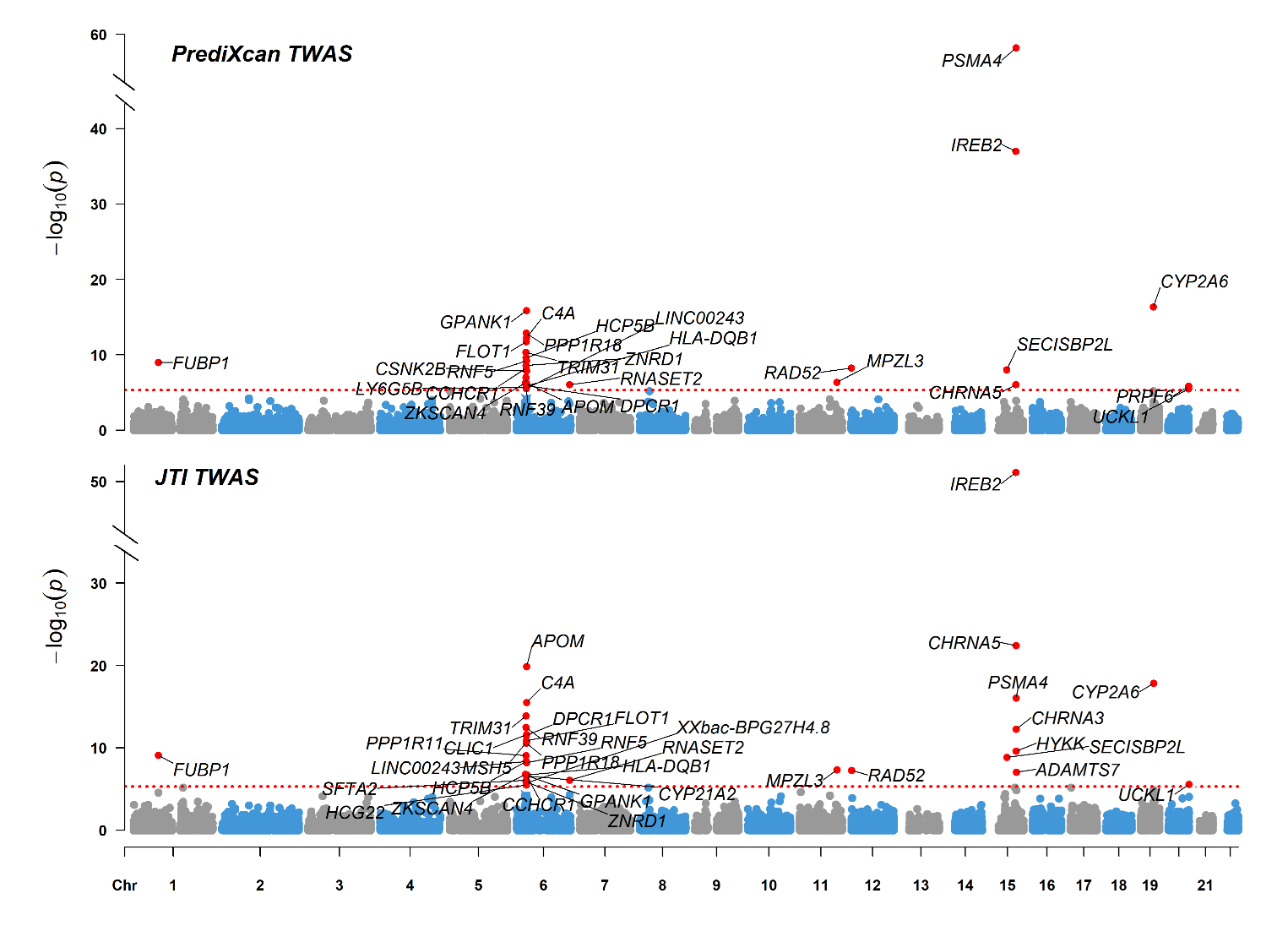


**Supplementary Figure 2**. Bar plot of TWAS-identified genes significantly associated with risk for lung cancer overall and histological subtypes.


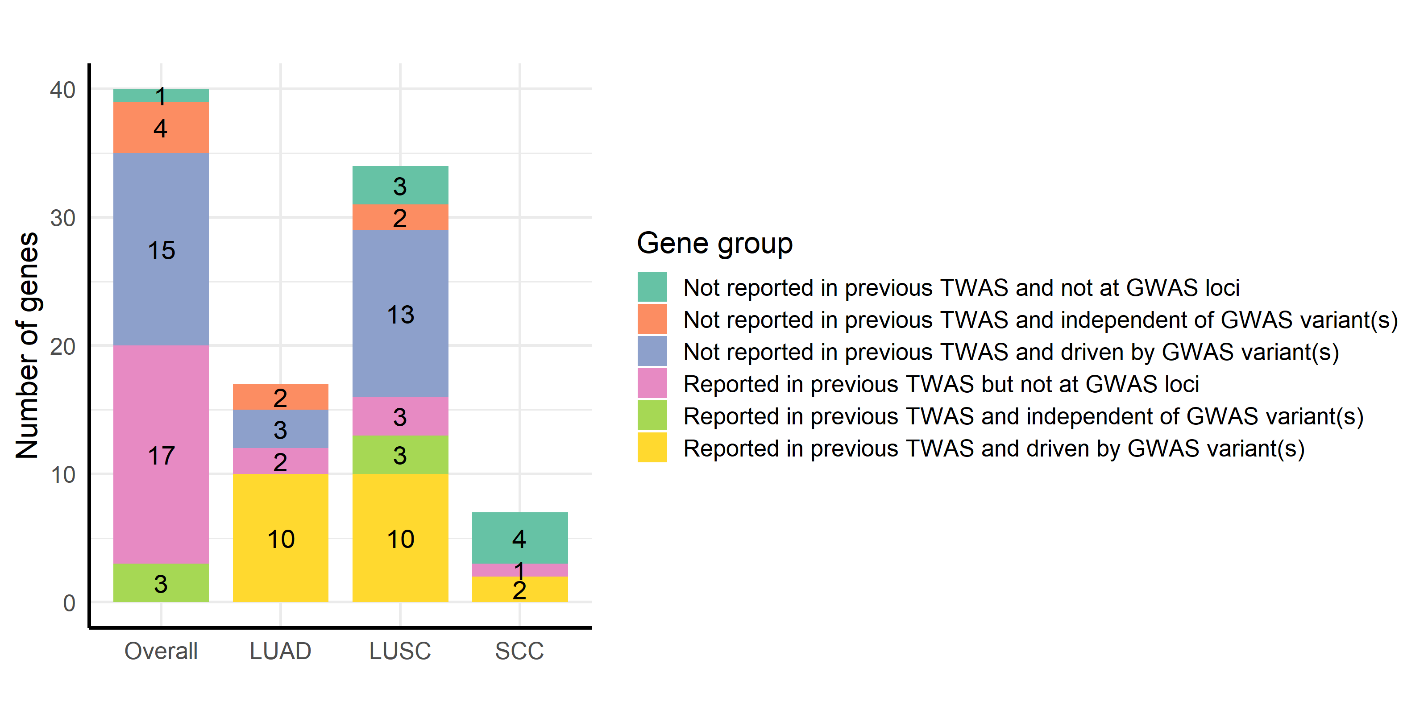


**Supplementary Figure 3**. Performance of thirteen genes located at genomic loci at least 2 Mb away from any GWAS-identified lung cancer risk variants in all lung cancer types. The significant heatmap cells are noted by Bonferroni corrected *p*-values < 0.05 with stars and the directions of associations between genes and lung cancer risk are represented by colors, with red signifying positive Z-scores (i.e., betas/standard errors) and blue signifying negative Z-scores.


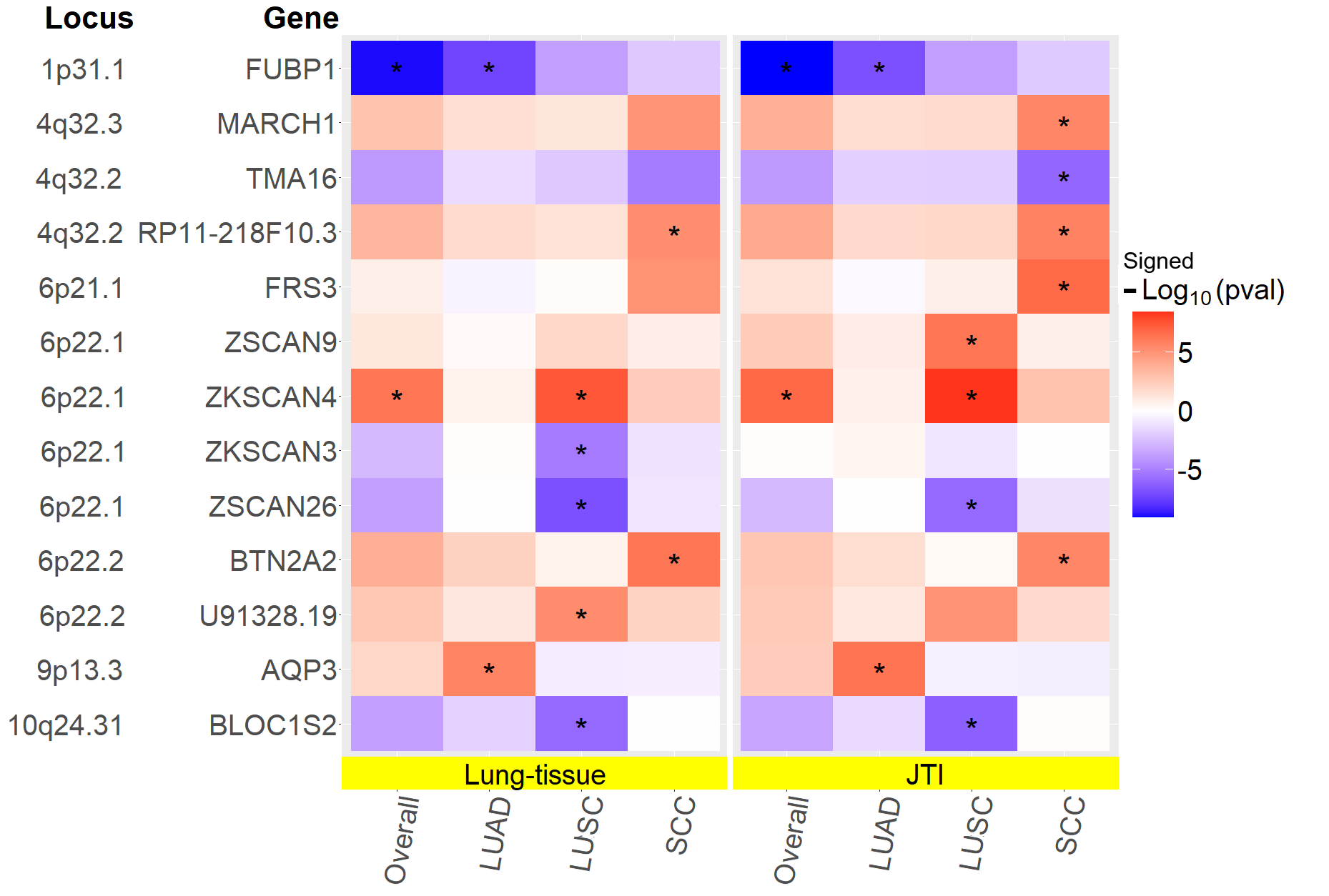

Supplement: Supplementary file 1 — Table S1: Number of genes with model building performance p < 0.05. Table S2: Performance of 13 genes located at genomic loci at least 2 Mb away from any GWAS‐identified lung cancer risk variants in all types. Table S3: Enrichr‐identified nominally enriched biological pathways with the 55 candidate risk genes for lung cancer. Figure S1: TWAS results for overall lung cancer. Figure S2: Bar plot of TWAS‐identified genes significantly associated with risk for lung cancer overall and histological subtypes. Figure S3: Performance of 13 genes located at genomic loci at least 2 Mb away from any GWAS‐identified lung cancer risk variants in all lung cancer types. [file CAM4-14-e71301-s001.docx]
